# Supplementary material for: Enantiodivergent Synthesis of Benzoquinolizidinones from L-Glutamic Acid
Source: Molecules. 2021 Sep 28;26(19):5866. doi: 10.3390/molecules26195866 (PMC8512815; doi:10.3390/molecules26195866)

molecules

Supplementary Information

Enantiodivergent Synthesis of Benzoquinolizidinones from L-glutamic Acid

Punlop Kuntiyong *,Duangkamon Namborisut, Kunita Phakdeeyothin, Rungrawin Chatpreecha and Kittisak Thammapichai

Department of Chemistry, Faculty of Science, Silpakorn University, Muang Nakhon Pathom 73000, Thailand; duangkamon177@gmail.com (D.N.); kunita.phakdeeyothin@gmail.com (K.P.); chatpreecha_R@su.ac.th (R.C.); kittisakthammapichai@gmail.com (K.T.)

* Correspondence: kuntiyong_p@su.ac.th; Tel.: +66-8-2569-9902

^{1}H and ^{13}C NMR Spectra of Compounds synthesized in this study (**11**, **12**, **14**, **15**, **17**, *ent*-**15**, **18**, **19**, **21**, **22**, **24**, *ent*-**22**, **26** and **28**)

(3*S*,5*R*)-1-(3,4-Dimethoxyphenylethyl)-3-(dibenzylamino)- 5-((*R*)-1-hydroxyethyl)piperidine-2,6-dione TBS ether **11**

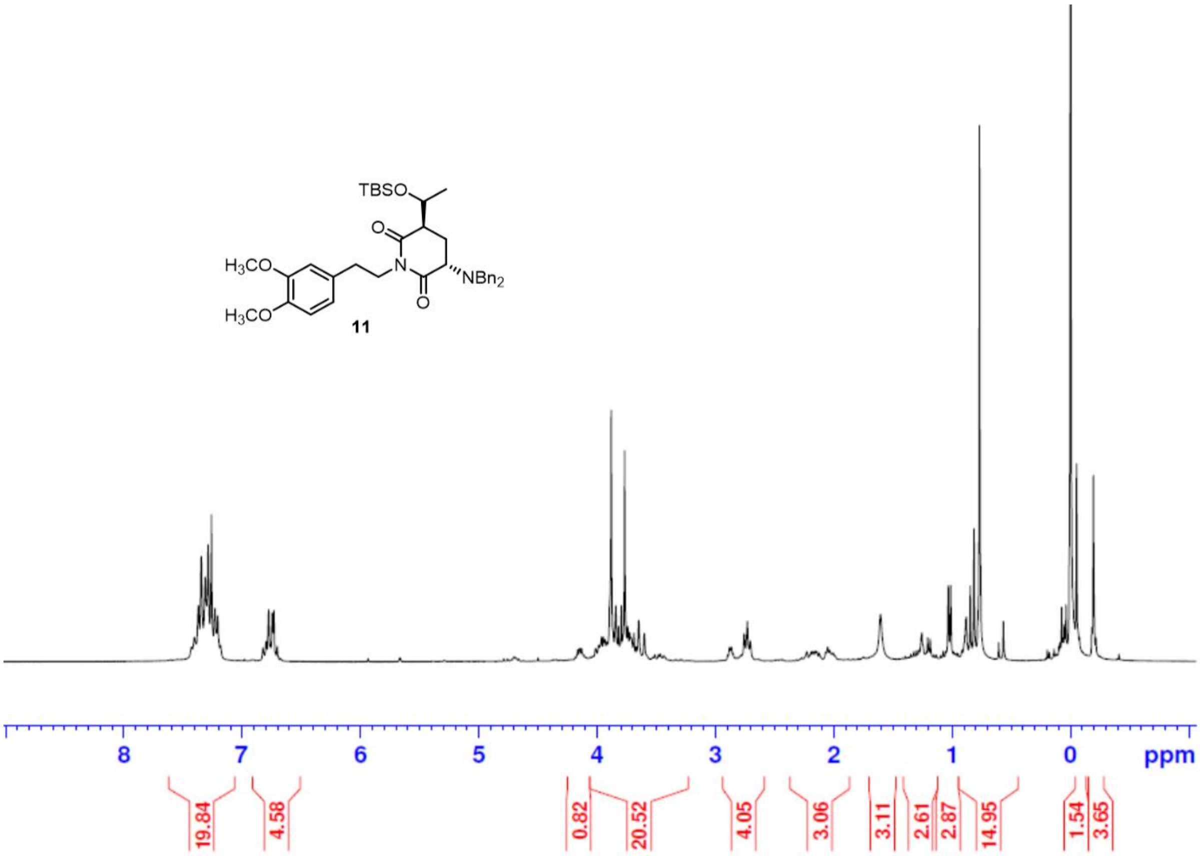

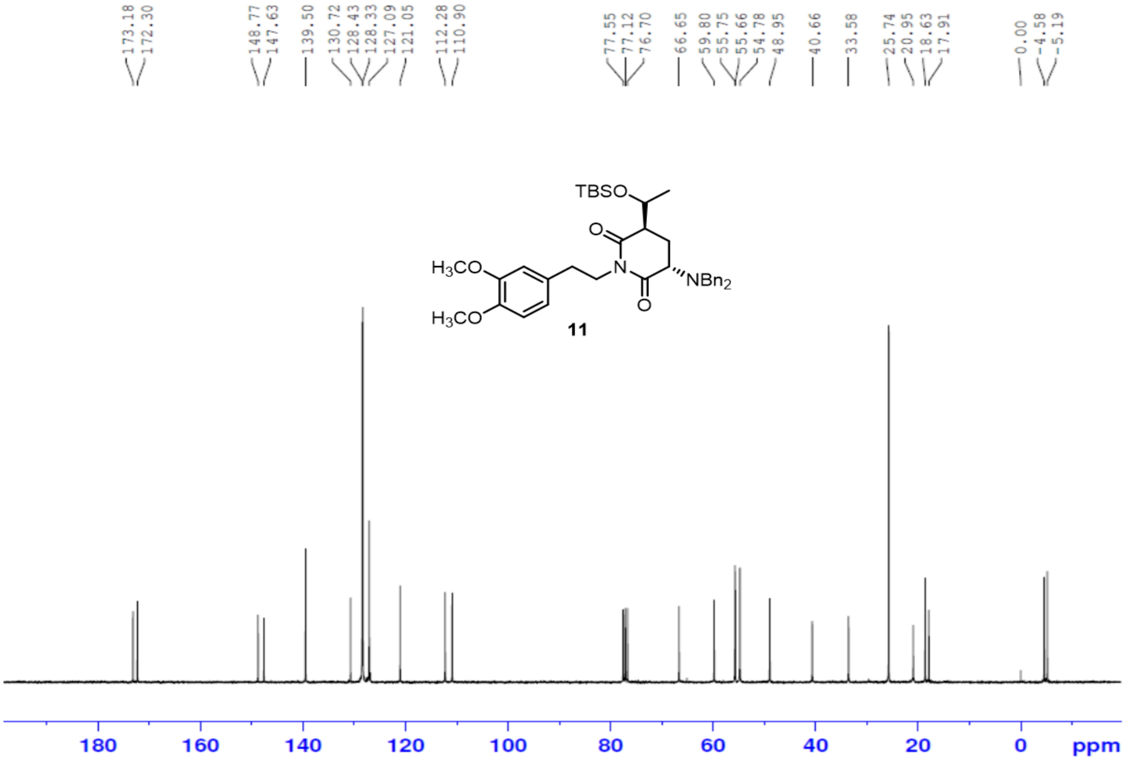

(3*S*,5*S*)-1-(3,4-Dimethoxyphenylethyl)-3-(dibenzylamino)- 5-((*S*)-1-hydroxyethyl)piperidine-2,6-dione TBS ether **12**

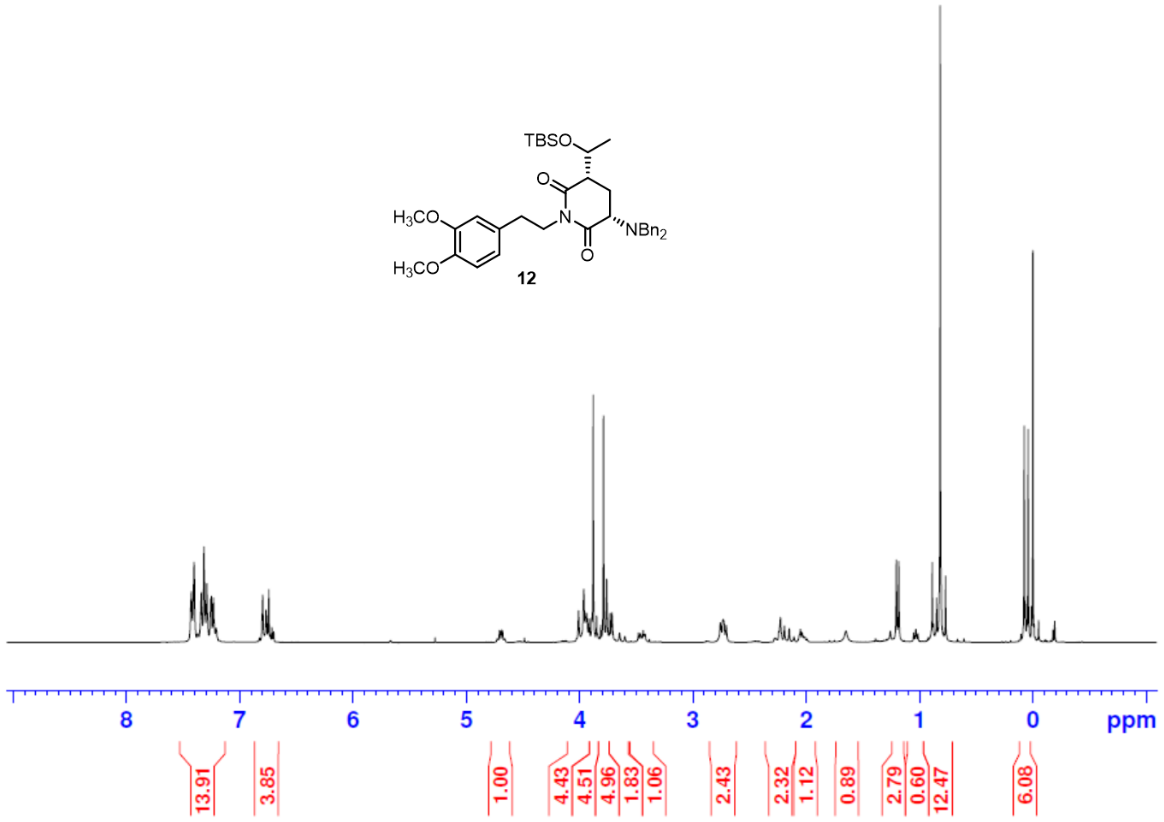

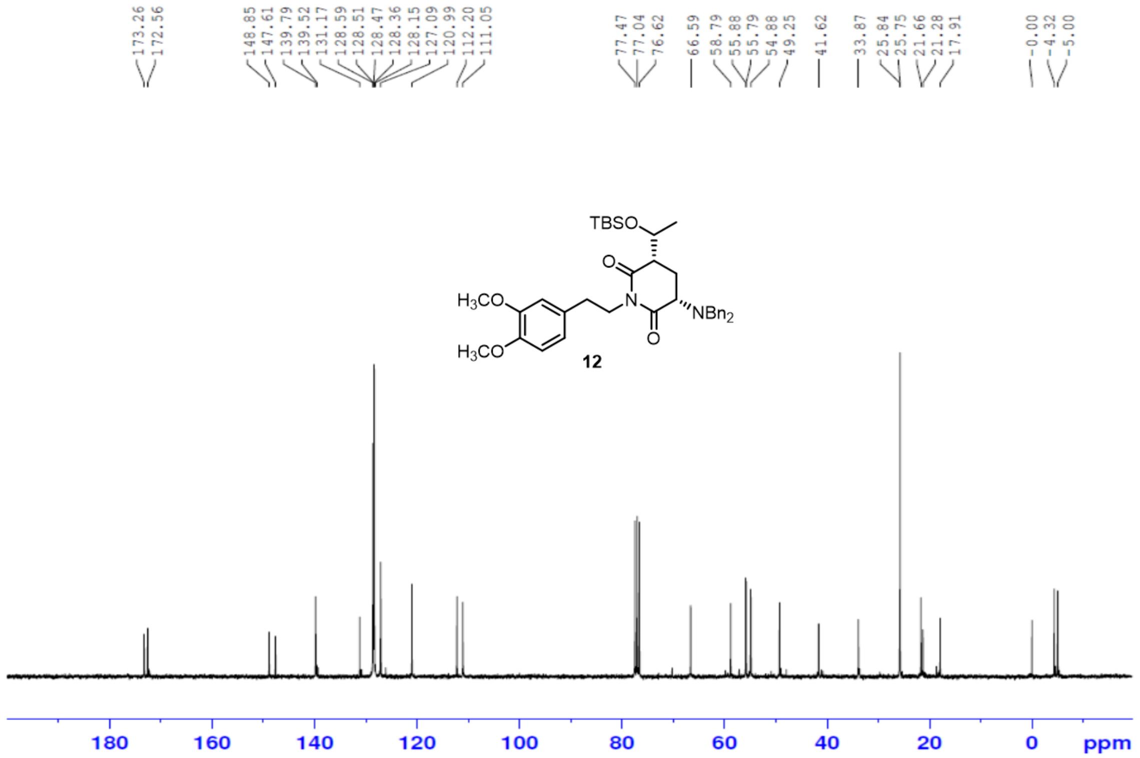

(1*S*,3*R*,11b*R*)-1-(dibenzylamino)-2,3,6,7-tetrahydro-3-((*S*)-1-hydroxyethyl)-9,10-dimethoxy-1*H*-pyrido[2,1-*a*]isoquinolin-4(11b*H*)-one (**14**)

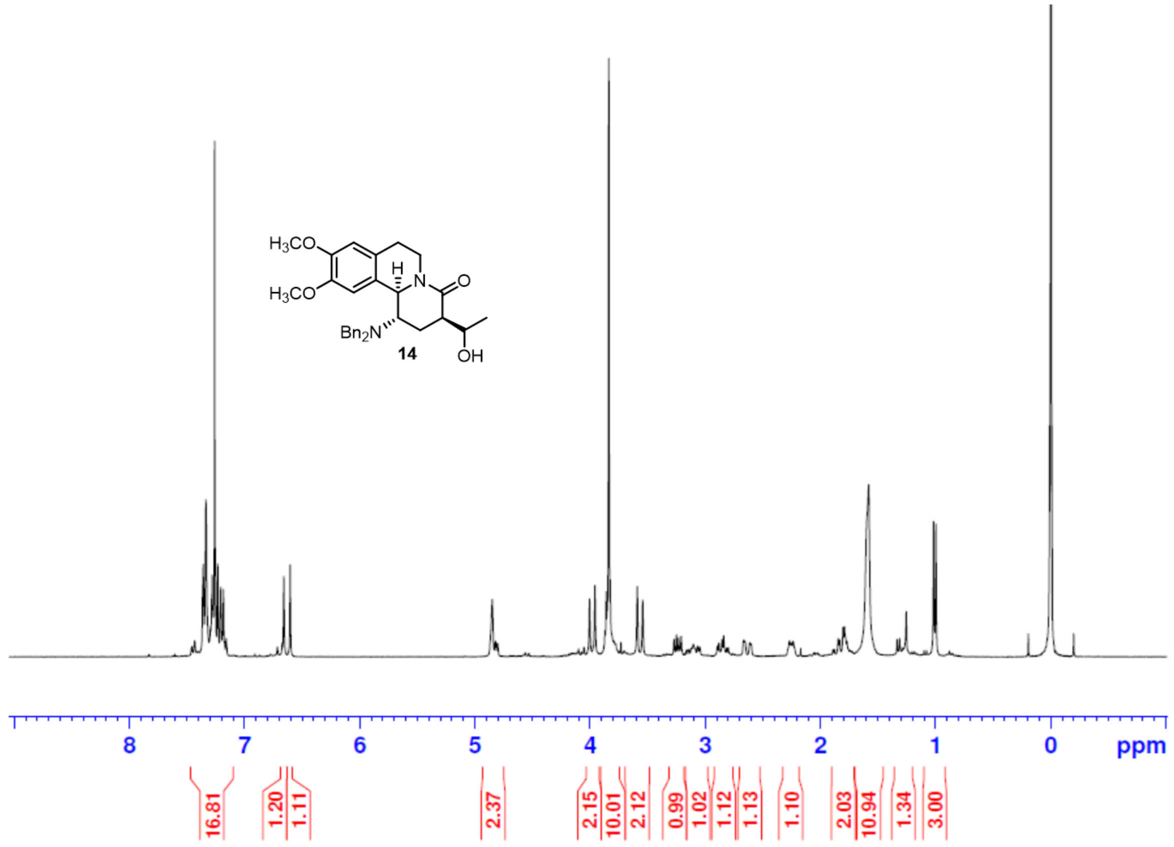

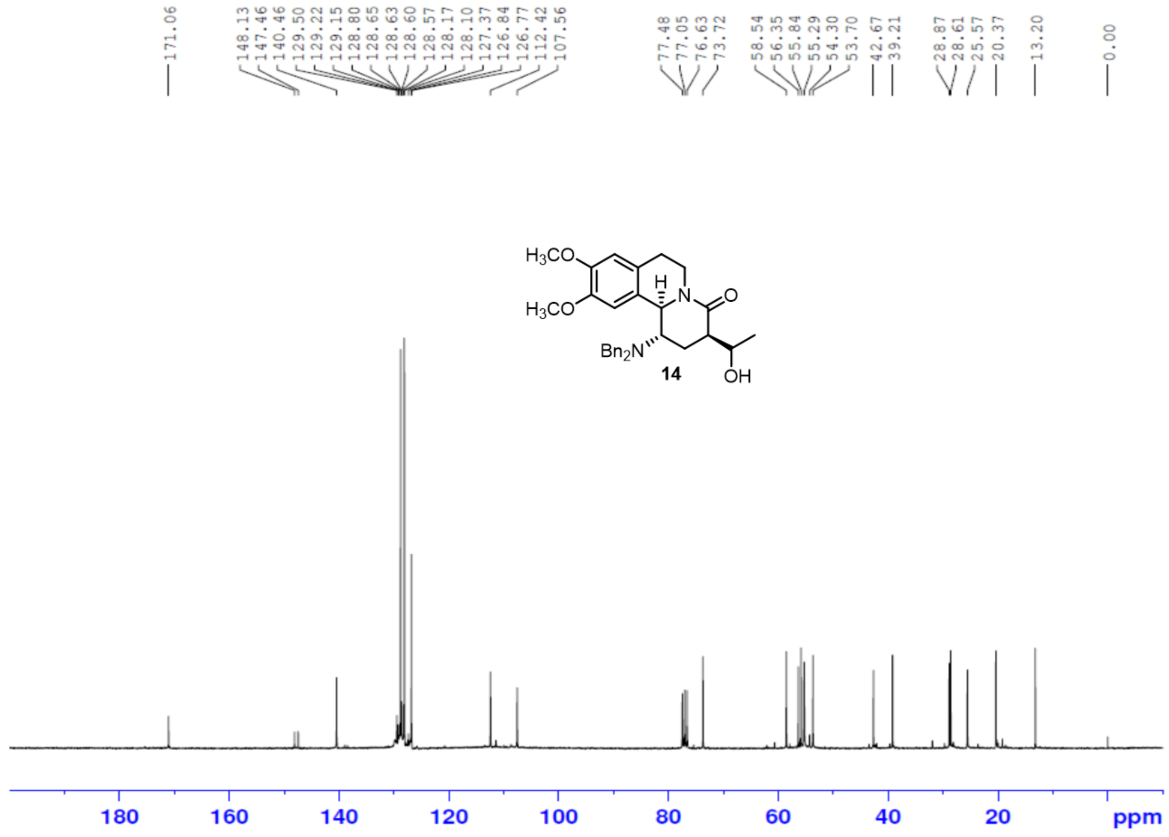

(3*R*,11b*S*)-6,7-dihydro-3-((*S*)-1-hydroxyethyl)-9,10-dimethoxy-3*H*-pyrido[2,1-*a*]isoquinolin-4(11b*H*)-one (**15**)

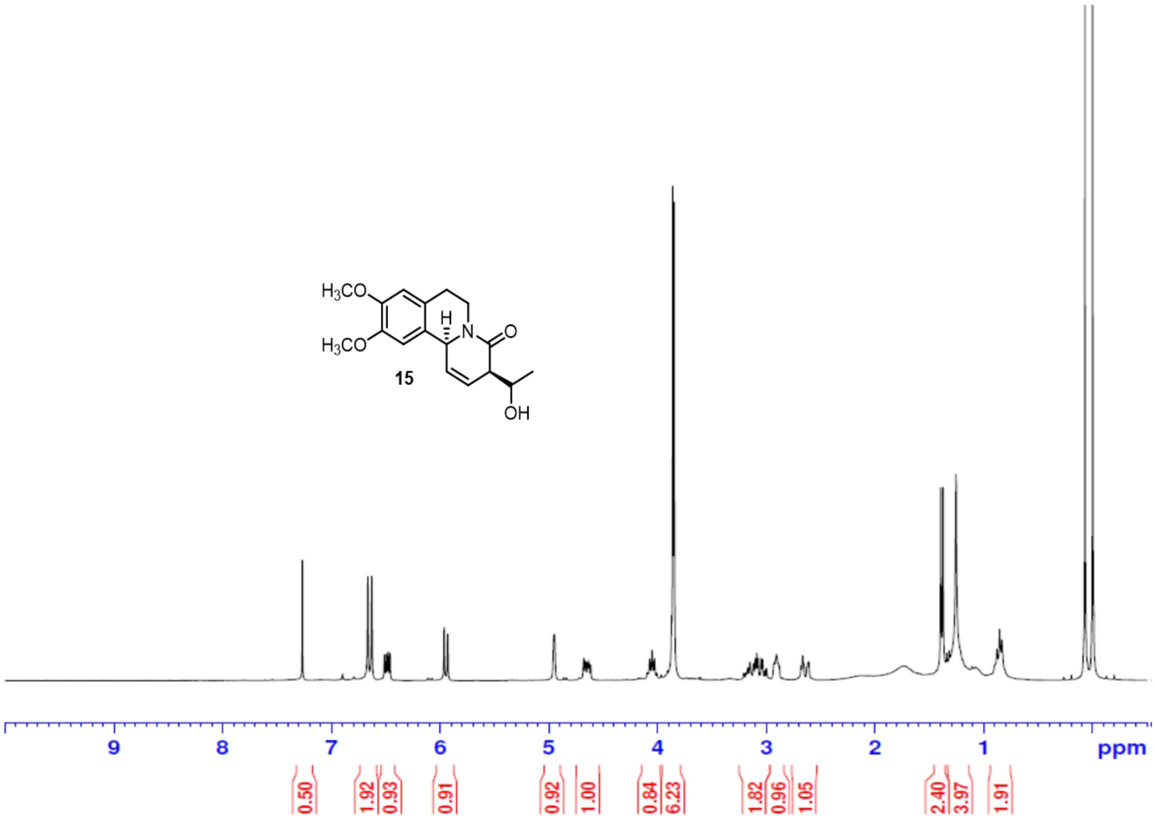

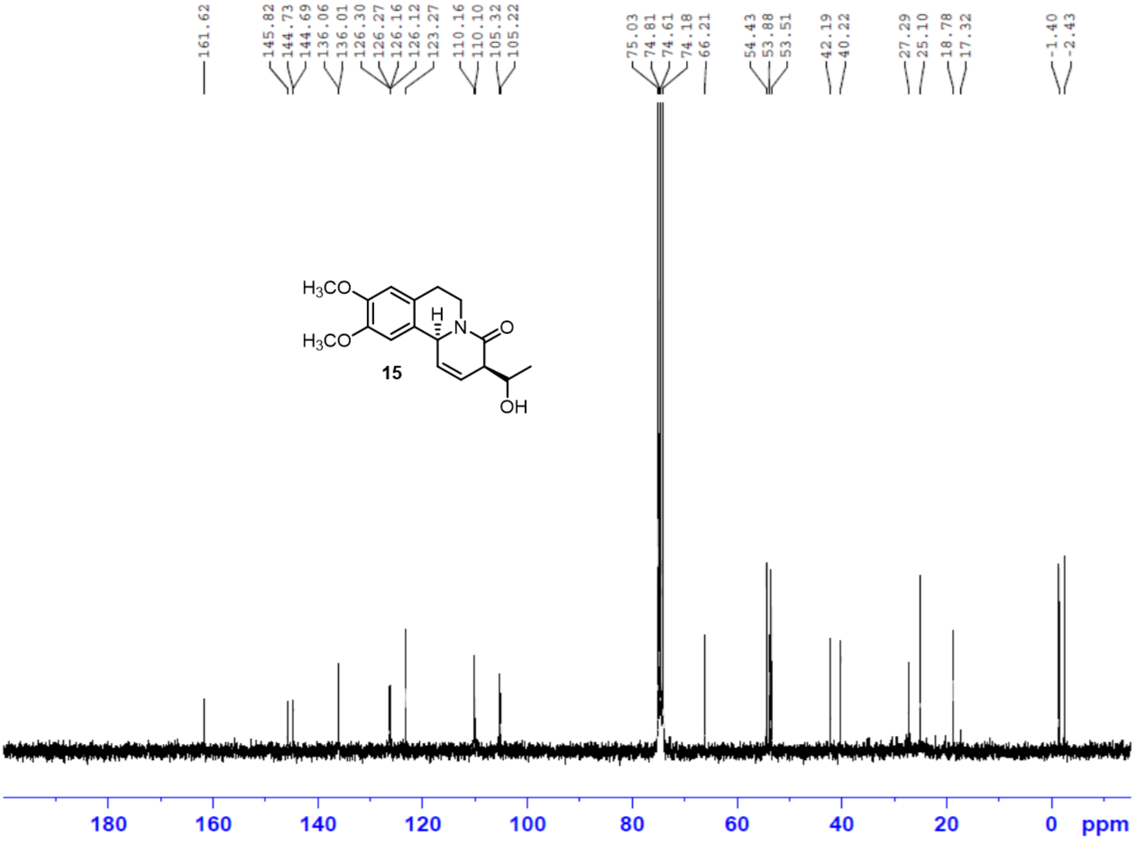

(1*S*,3*S*,11b*S*)-1-(dibenzylamino)-2,3,6,7-tetrahydro-3-((*S*)-1-hydroxyethyl)-9,10-dimethoxy-1*H*-pyrido[2,1-*a*]isoquinolin-4(11b*H*)-one (**17**)

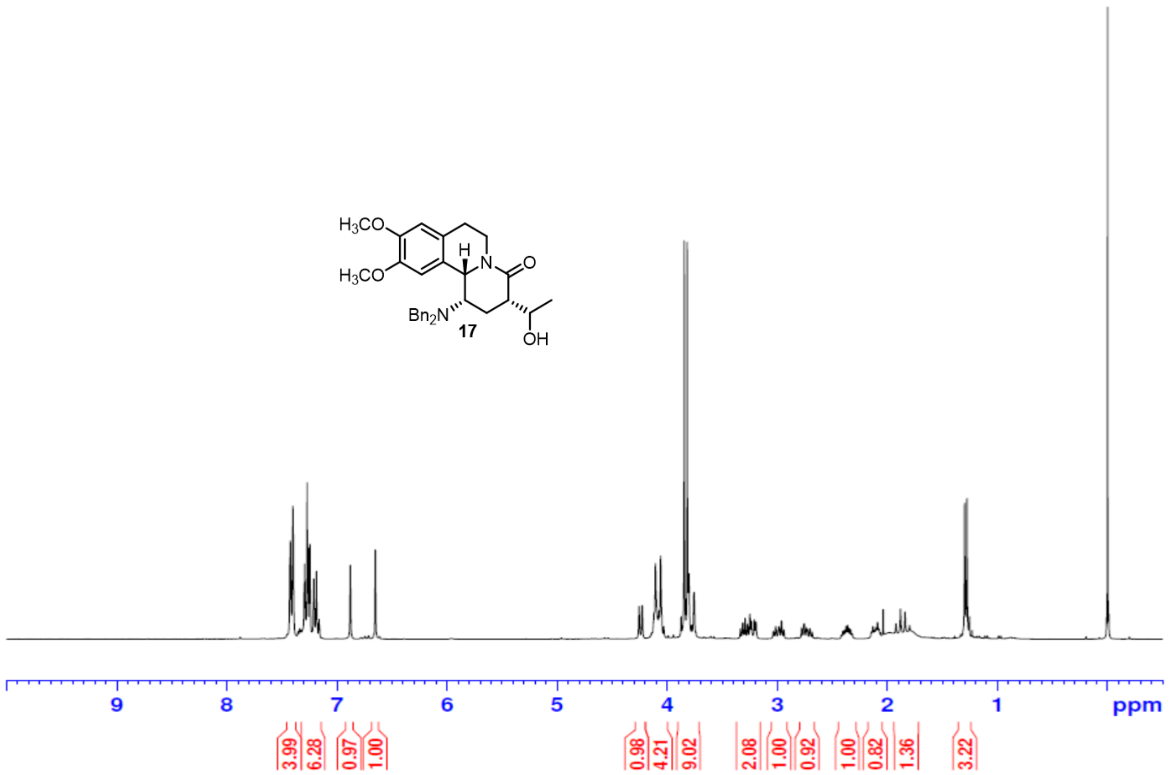

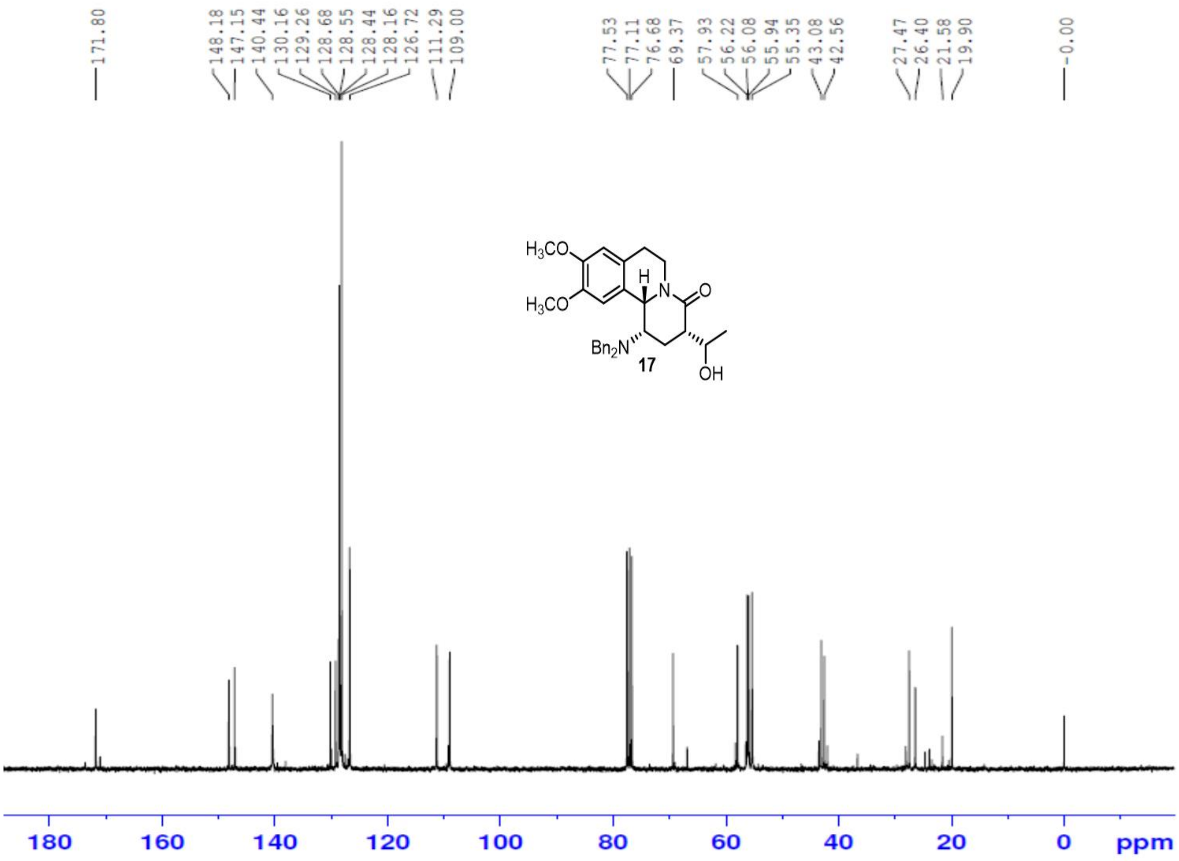

(3*S*,11b*R*)-6,7-dihydro-3-((*R*)-1-hydroxyethyl)-9,10-dimethoxy-3*H*-pyrido[2,1-*a*]isoquinolin-4(11b*H*)-one (*ent*-**15**)

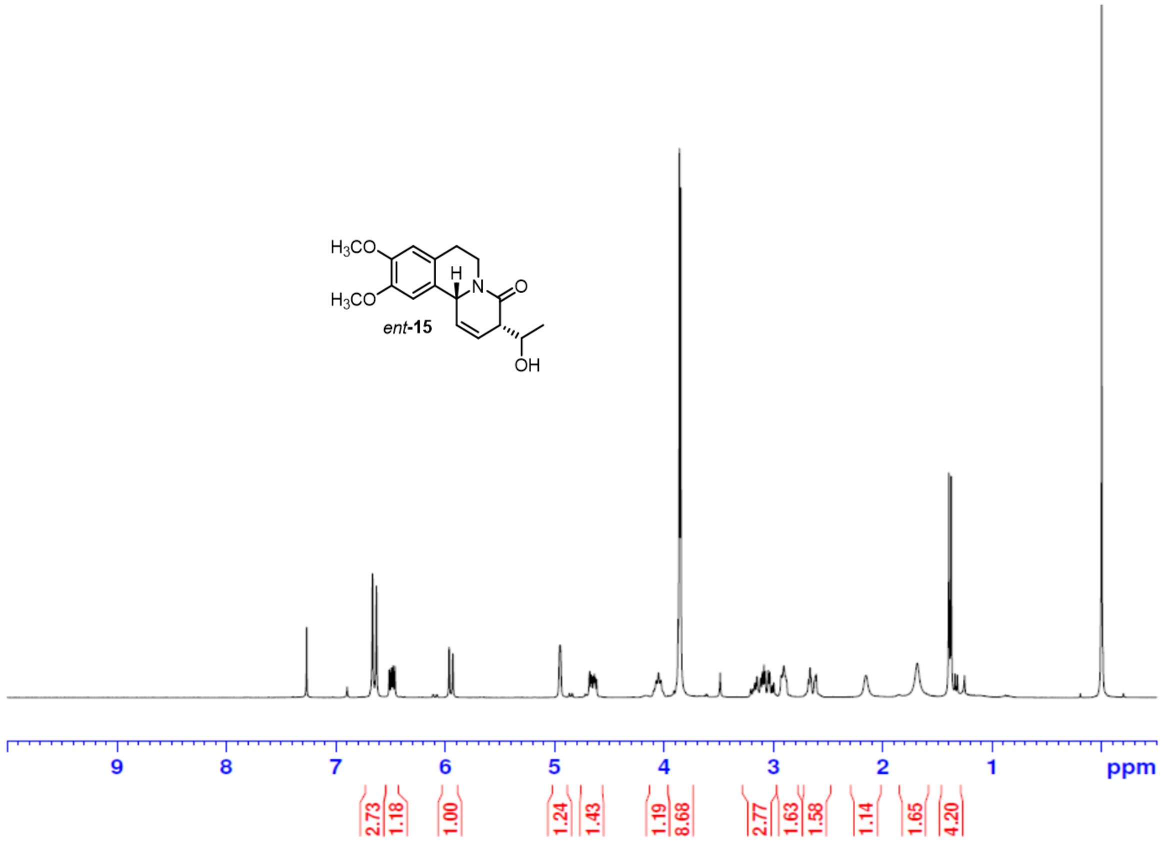

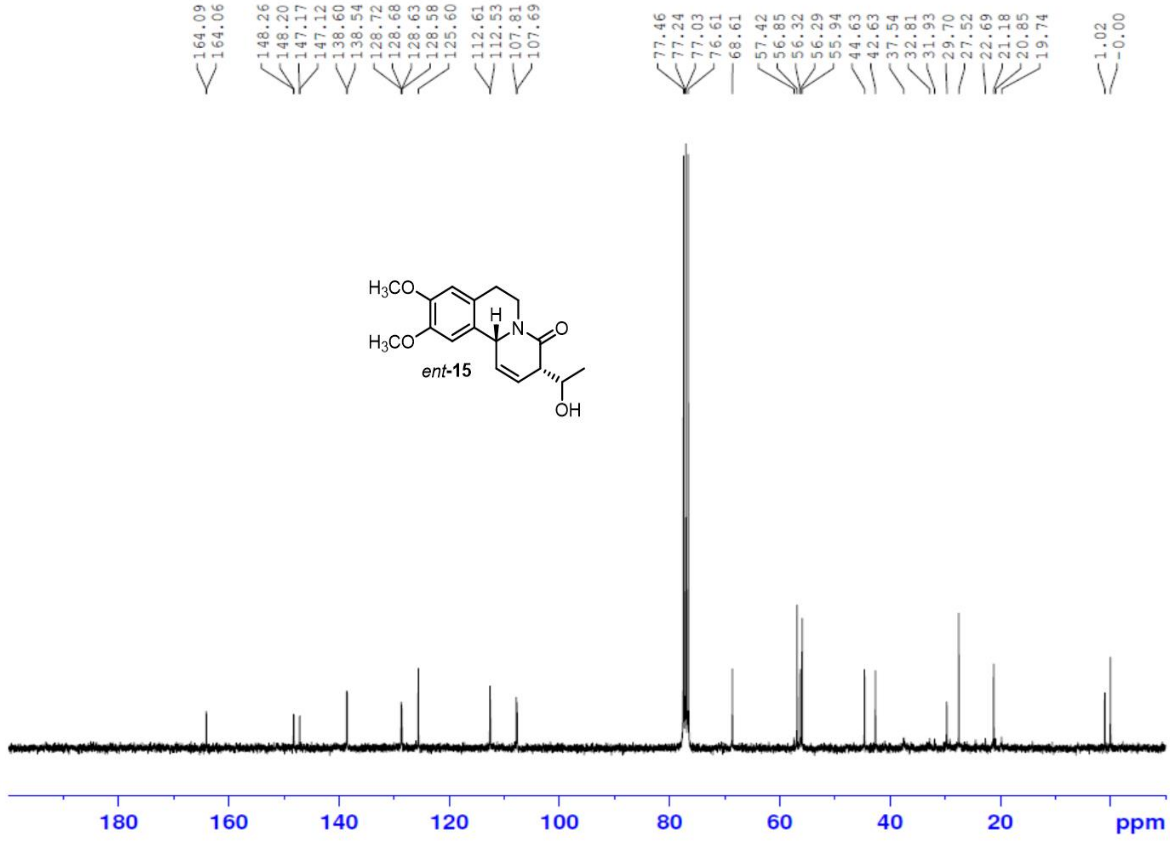

(3*S*,5*R*)-1-(3,4-Dimethoxyphenylethyl)-3-(dibenzylamino)- 5-((*S*)-1-hydroxy-2-methylpropyl)piperidine-2,6-dione (**18**)

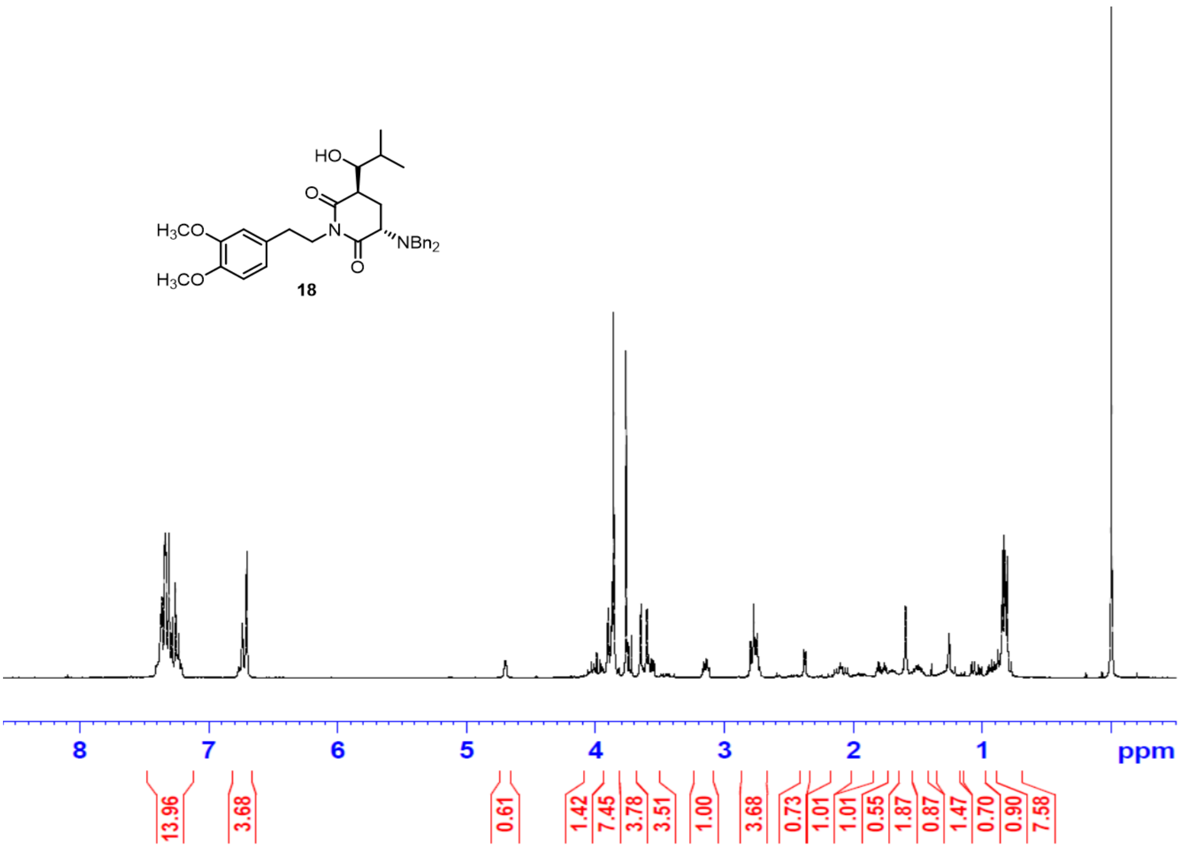

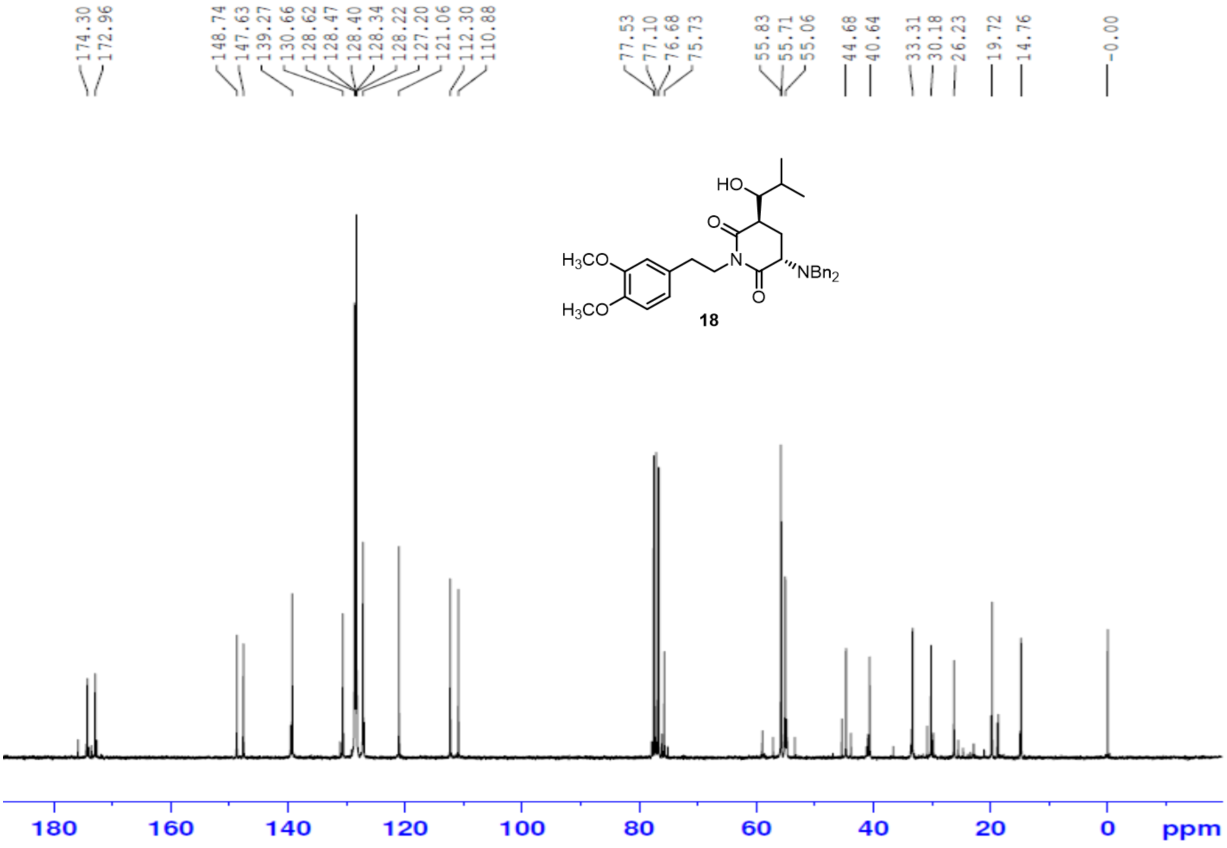

(3*S*, 5*S*)-1-(3,4-Dimethoxyphenylethyl)-3-(dibenzylamino)- 5-((*R*)-1-hydroxy-2-methylpropyl)piperidine-2,6-dione (**19**)

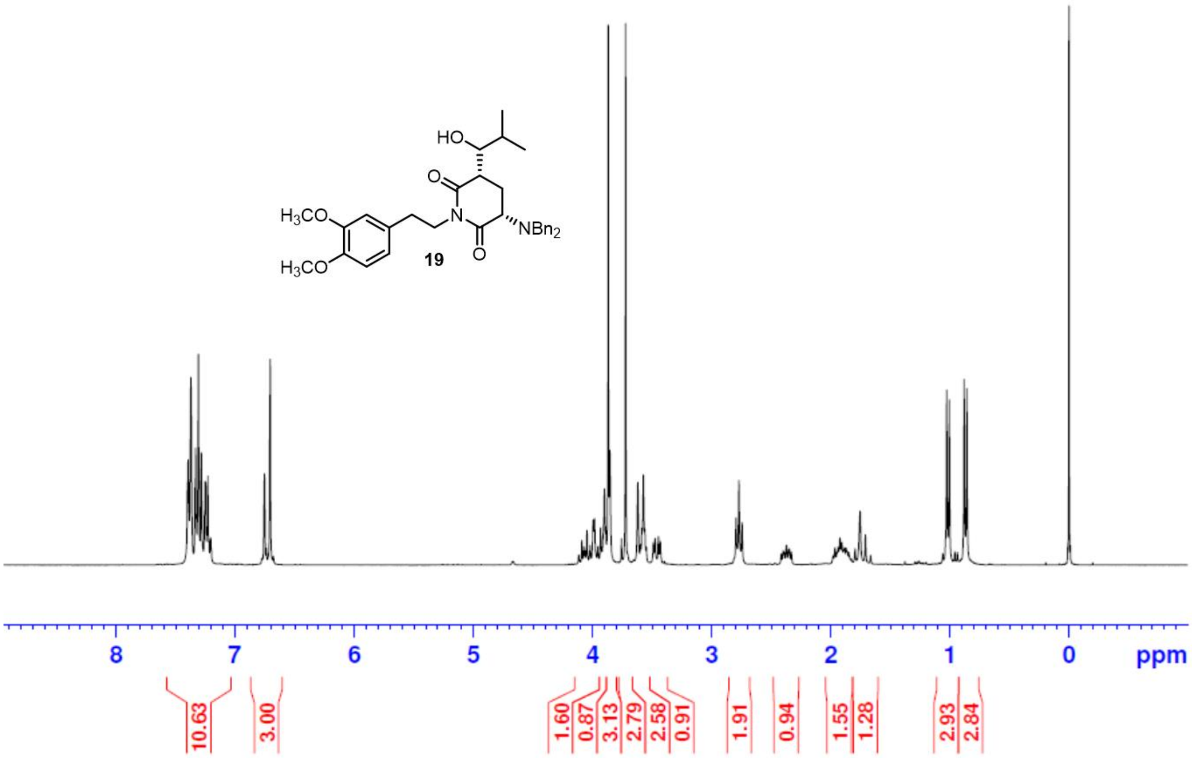

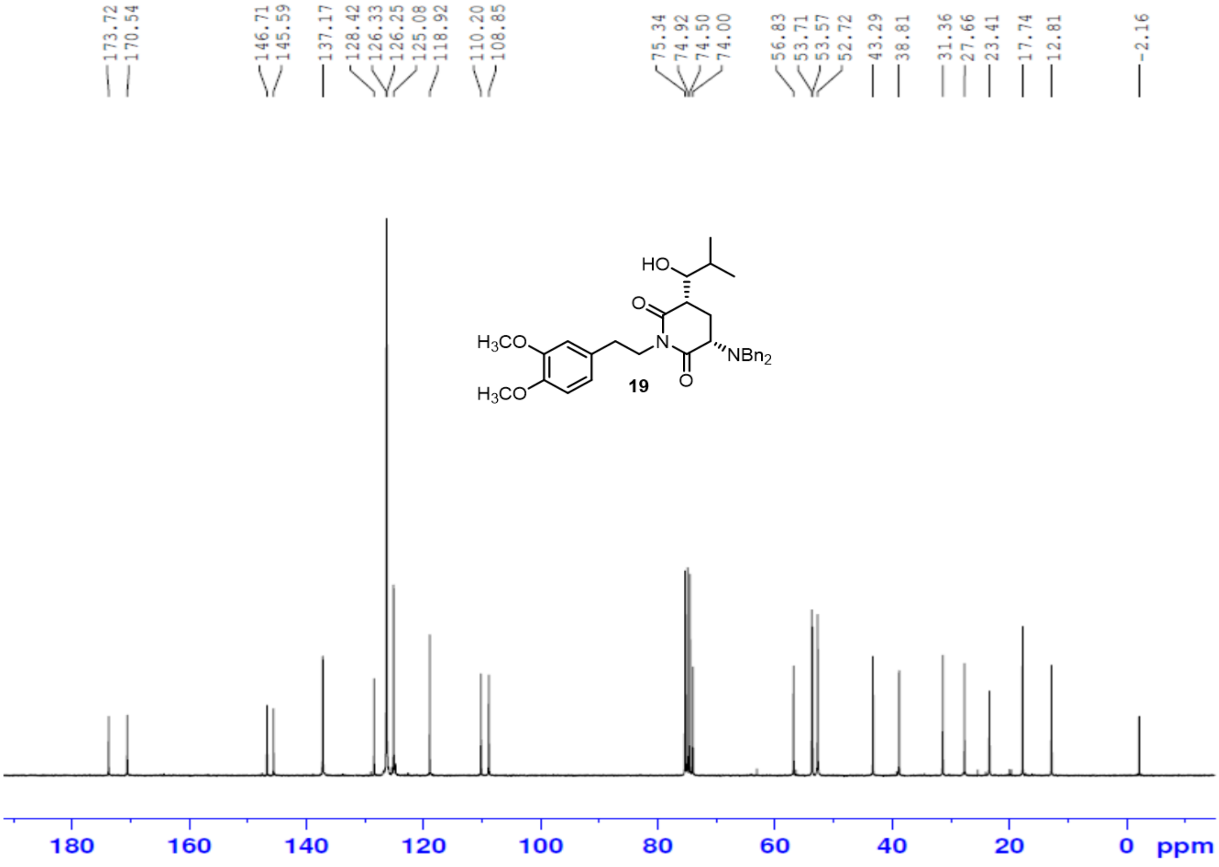

(1*S*,3*R*,11b*R*)-1-(dibenzylamino)-2,3,6,7-tetrahydro-3-((*S*)-1-hydroxy-2-methylpropyl)-9,10-dimethoxy-1*H*-pyrido[2,1-*a*]isoquinolin-4(11b*H*)-one (**21**)

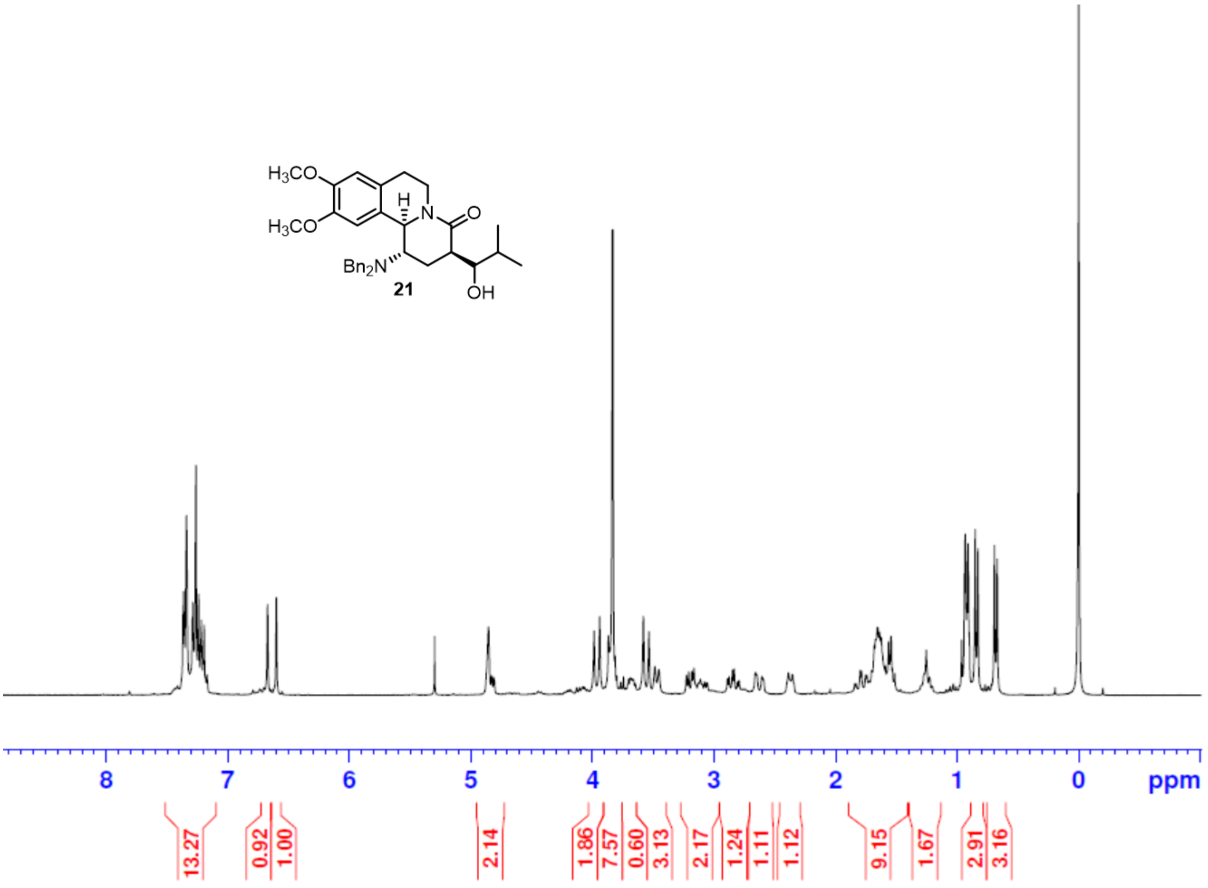

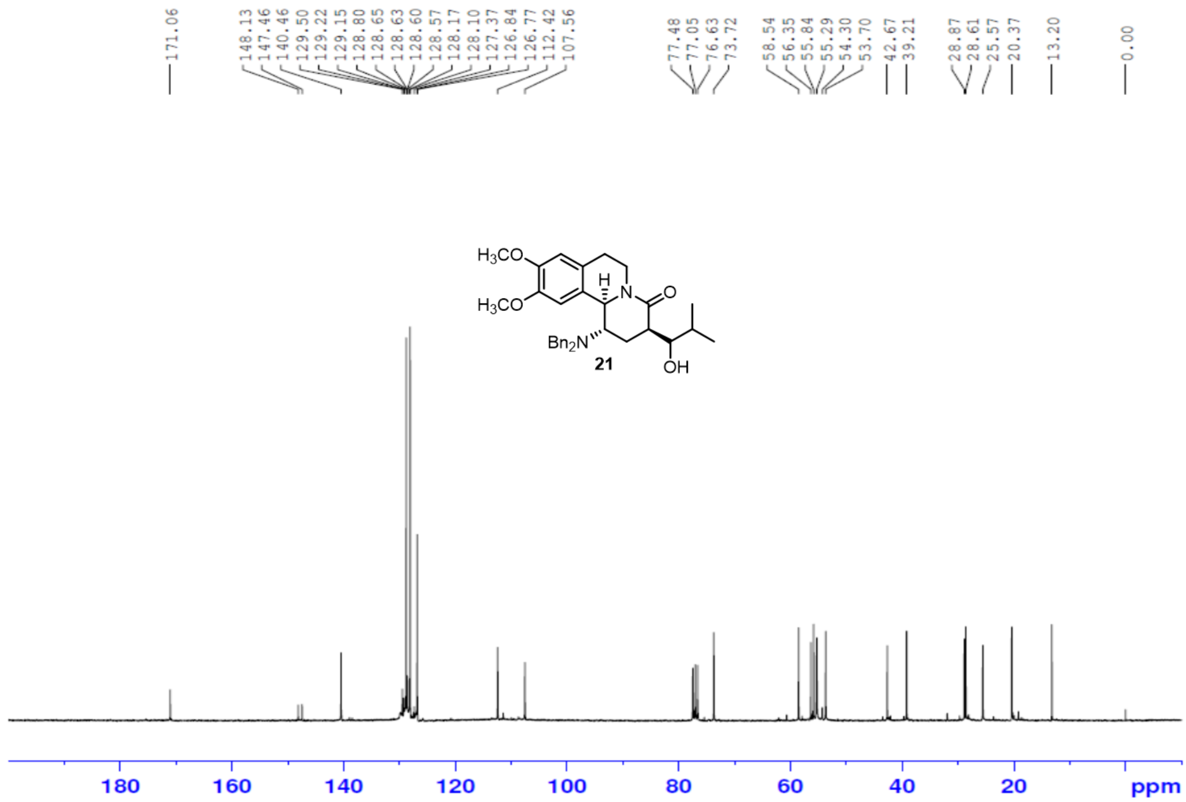

(3*R*,11b*S*)-6,7-dihydro-3-((*S*)-1-hydroxy-2-methylpropyl)-9,10-dimethoxy-3*H*-pyrido[2,1-*a*]isoquinolin-4(11b*H*)-one (**22**)

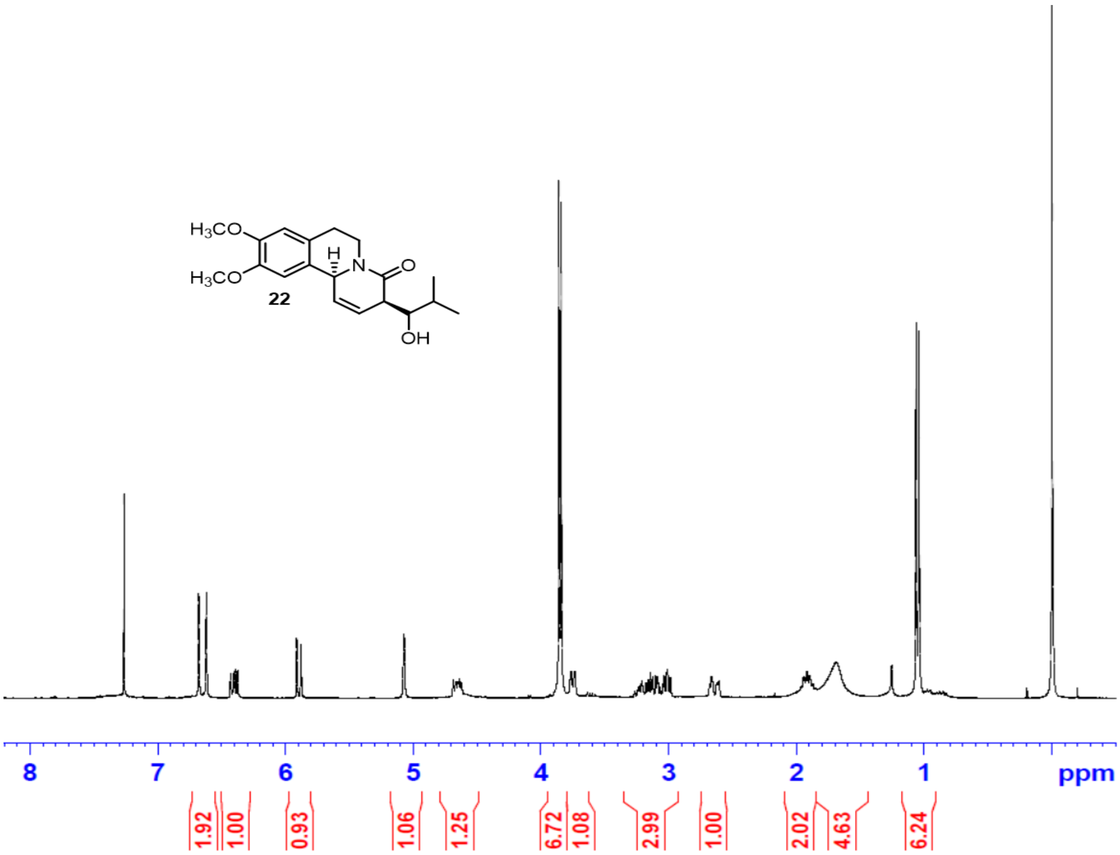

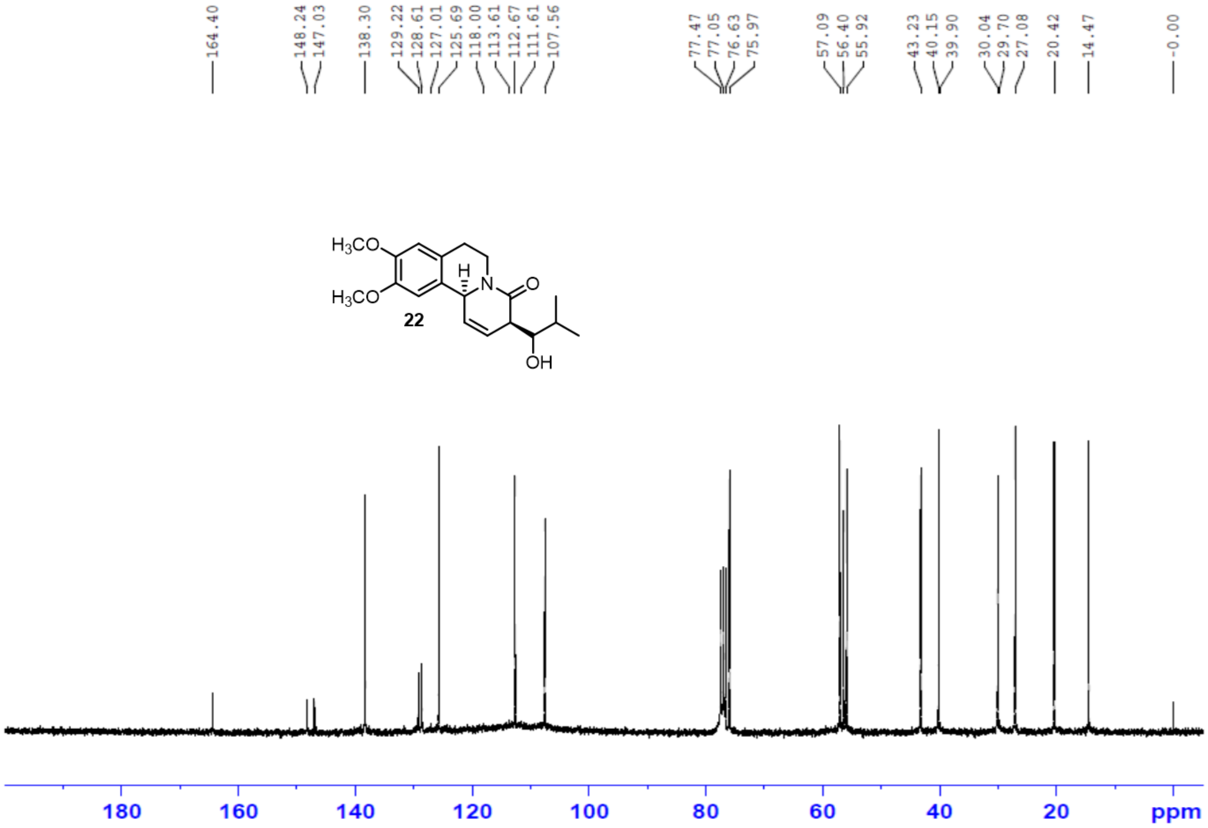

(1*S*,3*S*,11b*S*)-1-(dibenzylamino)-2,3,6,7-tetrahydro-3-((*R*)-1-hydroxy-2-methylpropyl)-9,10-dimethoxy-1*H*-pyrido[2,1-*a*]isoquinolin-4(11b*H*)-one (**24**)

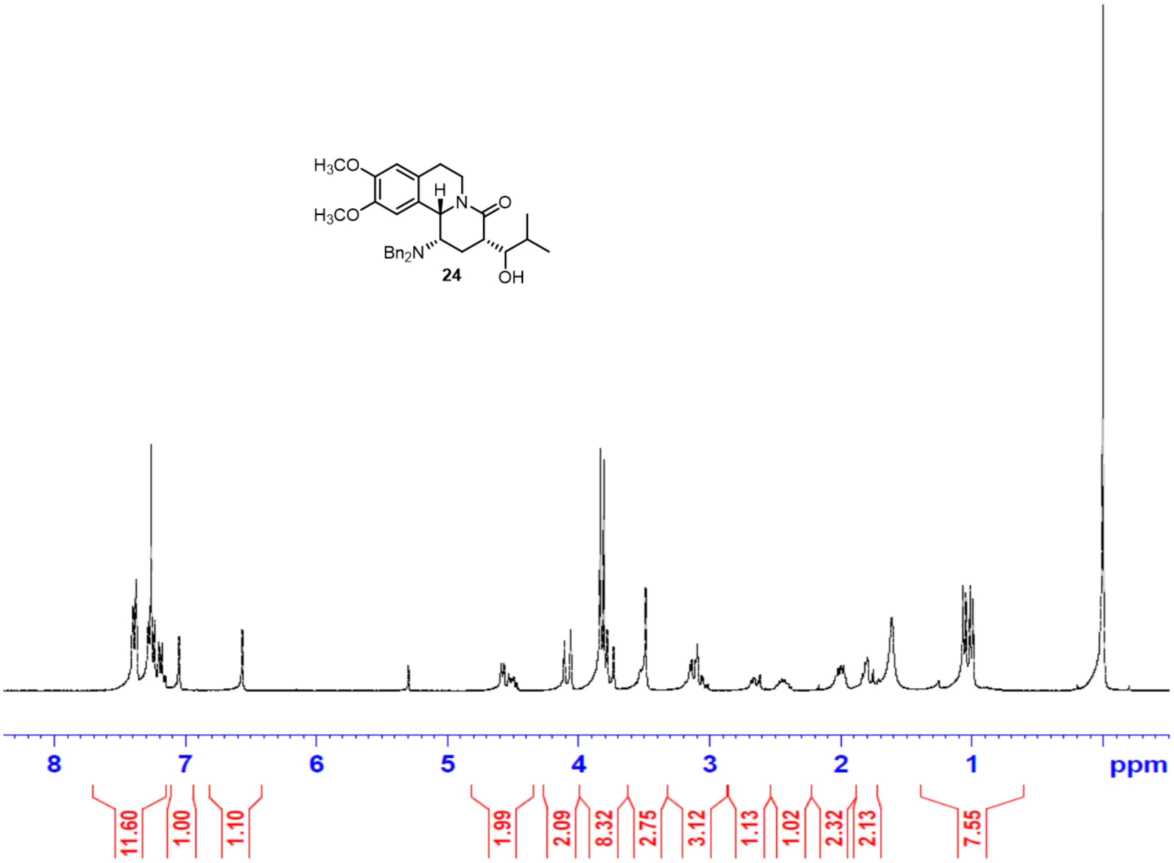

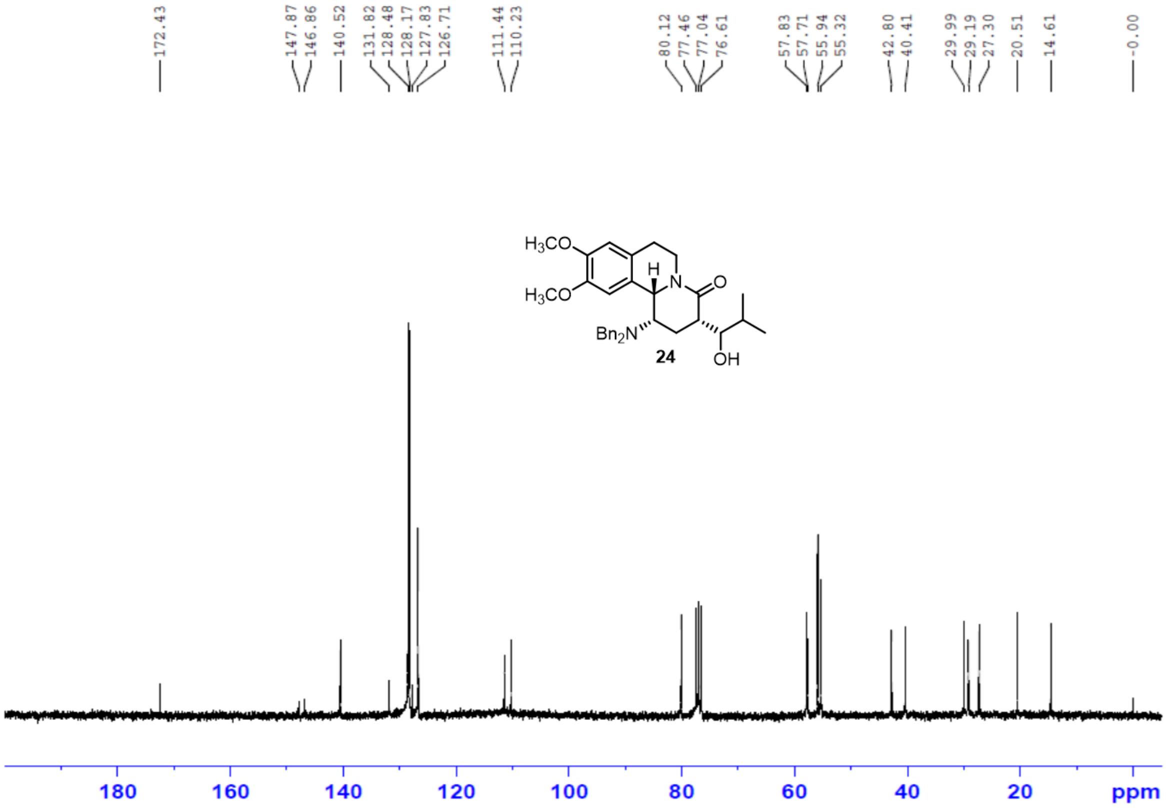

(3*S*,11b*R*)-6,7-dihydro-3-((*R*)-1-hydroxy-2-methylpropyl)-9,10-dimethoxy-3*H*-pyrido[2,1-*a*]isoquinolin-4(11b*H*)-one (*ent*-**22**)

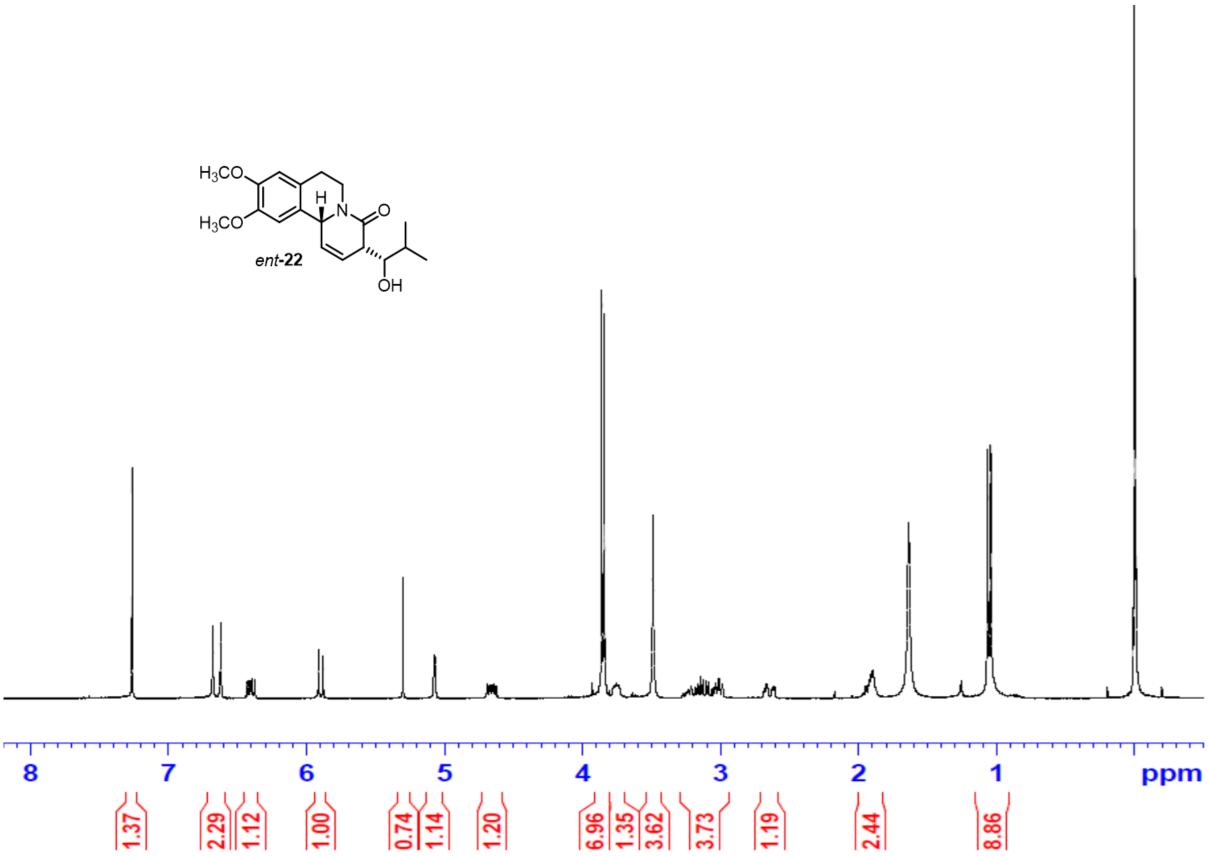

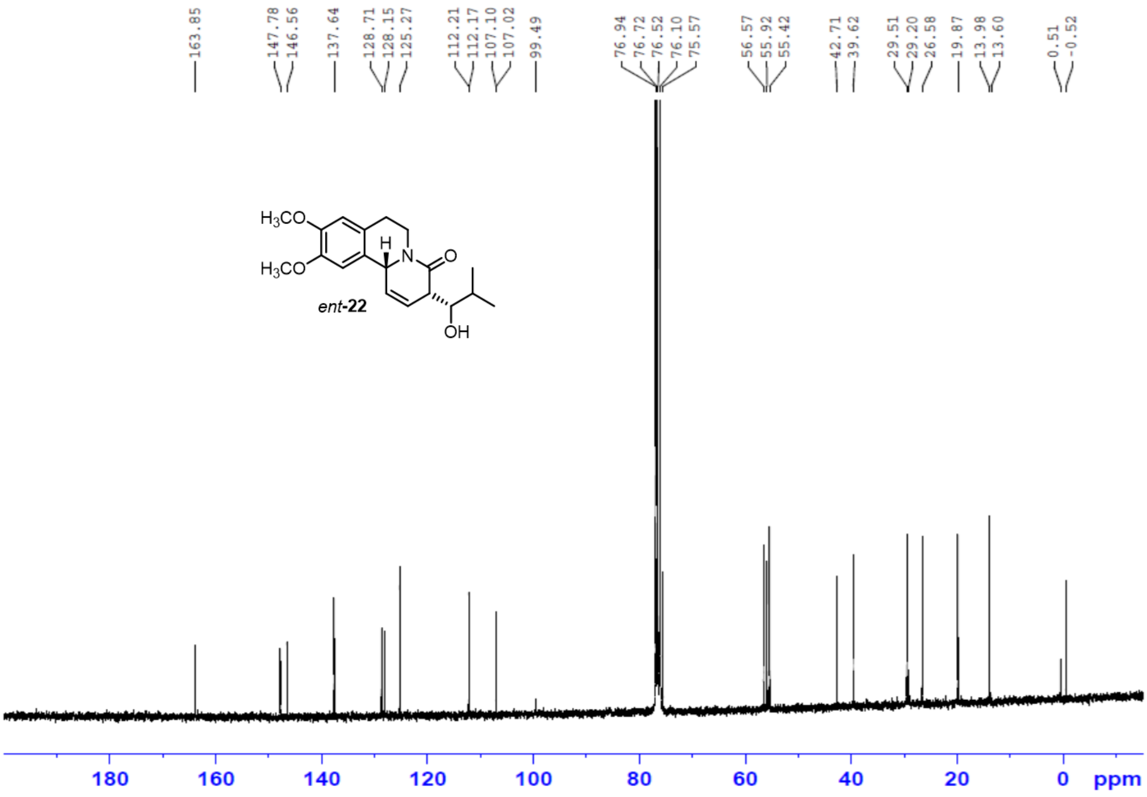

(3*S*, 5*S*)-1-(3,4-Dimethoxyphenylethyl)-3-(dibenzylamino)- 5-((*R*)-1-hydroxyethyl)piperidine-2,6-dione TBS ether **26**

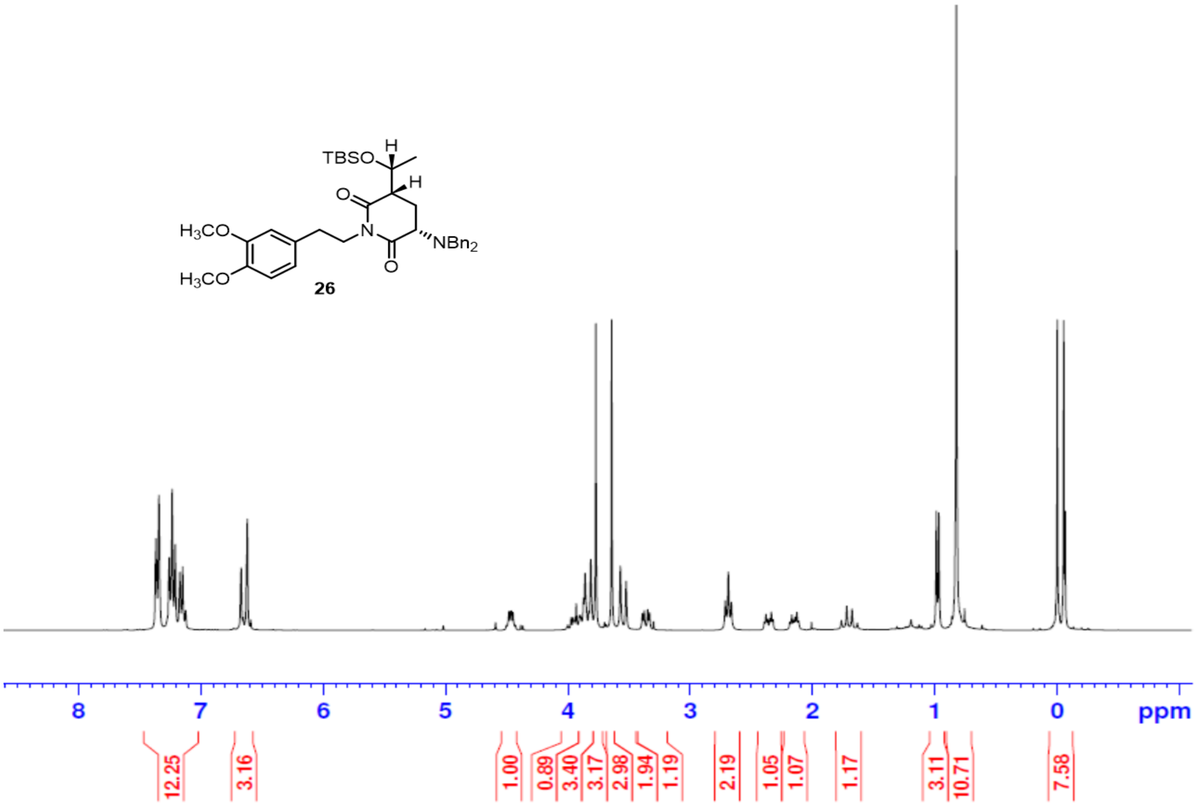

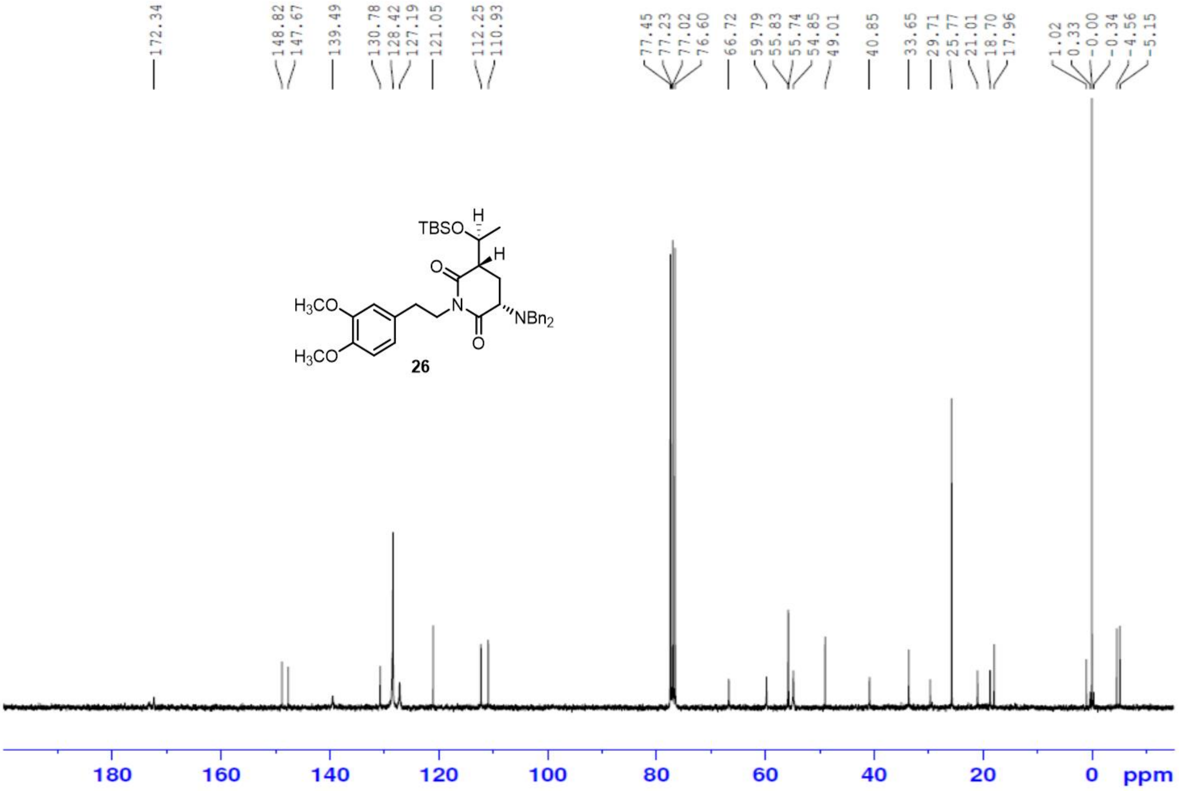

(1*S*,3*S*,11b*S*)-1-(dibenzylamino)-2,3,6,7-tetrahydro-3-((*R*)-1-hydroxyethyl)-9,10-dimethoxy-1*H*-pyrido[2,1-*a*]isoquinolin-4(11b*H*)-one (**28**)

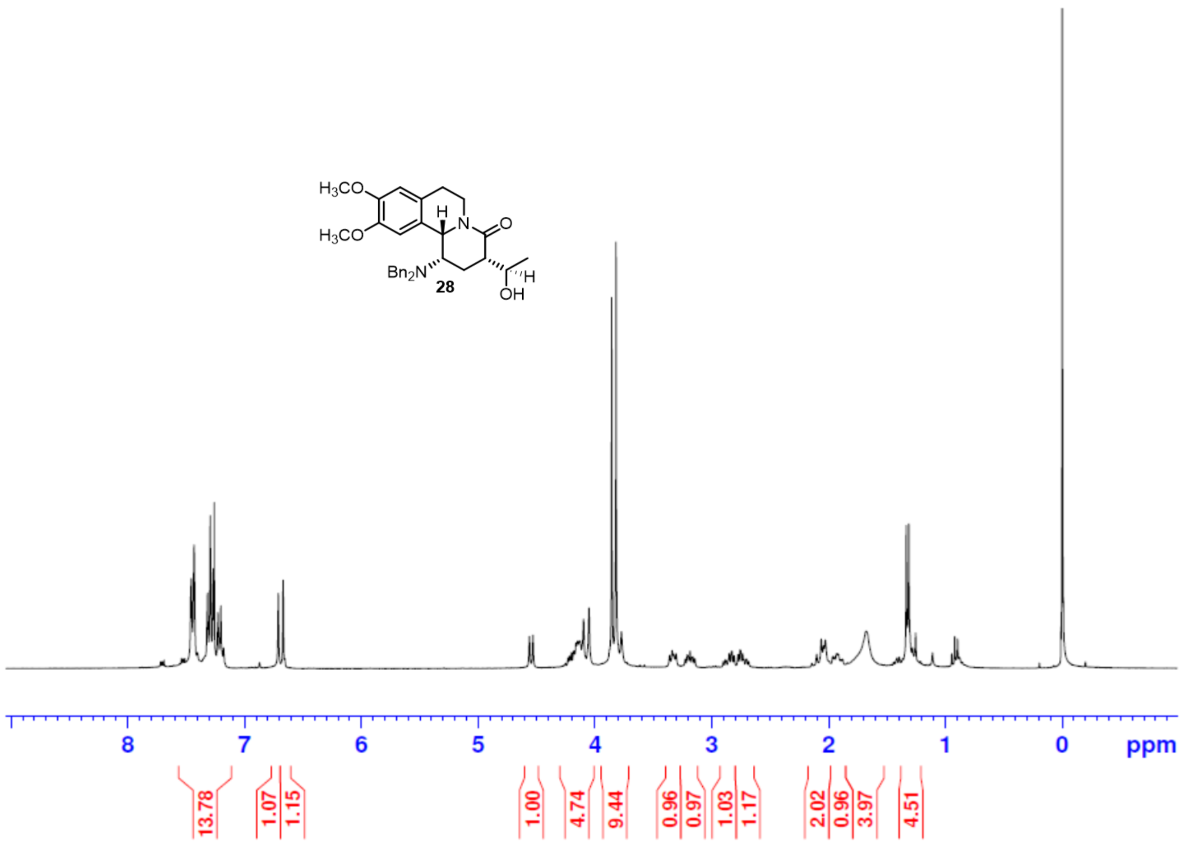

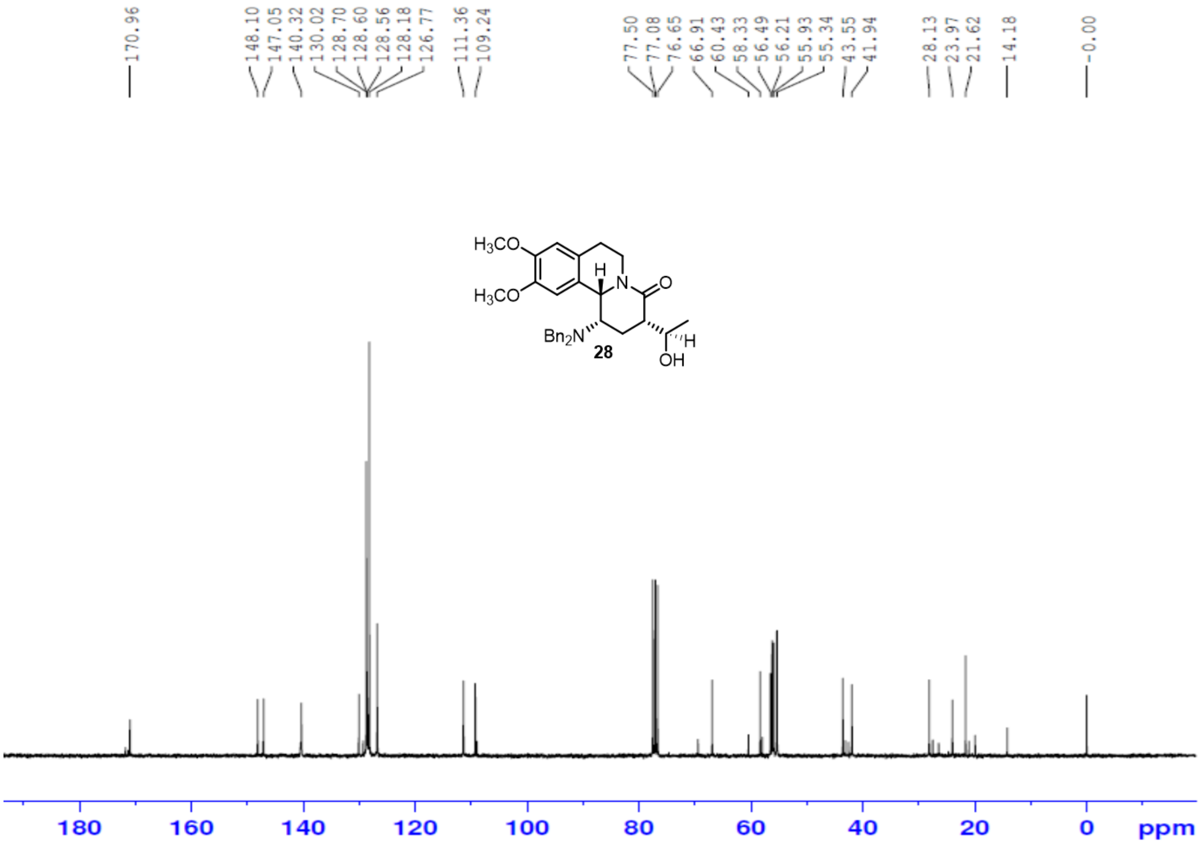

Supplement: Supplementary file 1 [file molecules-26-05866-s001.zip › molecules-1382573-supplementary.pdf]
